# Protein chip and bioinformatic analyses of differentially expressed proteins involved in the effect of hydrogen-rich water on myocardial ischemia-reperfusion injury

**DOI:** 10.7150/ijms.35984

**Published:** 2019-08-14

**Authors:** Liangtong Li, Tongtong Liu, Xiangzi Li, Xuanchen Liu, Li Liu, Shaochun Li, Zhilin Li, Yujuan Zhou, Fulin Liu

**Affiliations:** 1 School of Medicine, Hebei University, Baoding, 071000, China; 2 Affiliated Hospital of Hebei University, Baoding, 071000, China; 3 School of Chemistry, Hebei University, Baoding 071000, China; 4 Central Laboratory of Affiliated Hospital of Hebei University, Baoding 071000, China; 5 Department of Cardiac Surgery, Affiliated Hospital of Hebei University, Baoding, 071000, China

**Keywords:** hydrogen-rich water, myocardial ischemia-reperfusion injury, protein chip, GO enrichment analysis, KEGG pathway analysis

## Abstract

**Background**: The differentially expressed proteins (DEPs) involved in the effect of hydrogen-rich water on myocardial ischemia reperfusion injury (MIRI) and their biological processes and signaling pathway were analyzed. **Methods**: 20 Wistar rats were randomly and equally divided into a control and a hydrogen-rich group. Hearts were removed and fixed in a Langendorff device. The control group was perfused with K-R solution, and the hydrogen-rich water group was perfused with K-R solution + hydrogen-rich water. Protein was extracted from the ventricular tissues, and GSR-CAA-67 was used to identify the DEPs between two groups. DEPs were analyzed through bioinformatic methods. **Results:** Compared with the control group, in the treatment group, the expression of 25 proteins was obviously decreased (*P*<0.05). For the DEPs, 359 biological processes, including the regulation of signaling pathways, immune reaction and formation of cardiovascular endothelial cells, were selected by GO enrichment analysis. Five signaling pathways were selected by KEGG pathway enrichment analysis. **Conclusions:** 25 proteins that are involved in hydrogen-water reducing MIRI were selected by high-throughput GSR-CAA-67. The biological processes and metabolic pathways involved in the DEPs were summarized, providing theoretical evidence for the clinical application of hydrogen-rich water.

## 1. Introduction

Heart failure remains one of the most prevalent and challenging medical conditions with high morbidity and mortality despite advances in treatment 1-2. In recent years, with the advancement of shock treatment and the establishment and application of methods such as thrombolytic therapy, cardiopulmonary bypass, cardiopulmonary cerebral resuscitation, and organ transplantation, the blood circulation of myocardial tissue has been reconstructed, and the function is restored. Due to the repair of damaged tissue structure, the patients' condition can be improved or recovered. However, sometimes reperfusion after ischemia not only fails to restore the function of tissues and organs but also aggravates myocardial tissue, cardiac dysfunction and structural damage. This phenomenon of tissue damage and even irreversible damage after ischemic myocardial blood flow recovery is called myocardial ischemia-reperfusion injury (MIRI) 3-4. MIRI is the most common and serious pathophysiological phenomenon that occurs during the perioperative period that seriously affects the prognosis of patients 5, and methods to effectively treat this disease has attracted increasing attention.

As the study of MIRI increase, an increasing number of drugs are being discovered. In recent years, an element that has been overlooked has gradually become a focus of study: hydrogen; studies have found that hydrogen can attenuate MIRI 6-7. Liu Xue-cong 6 found that the use of hydrogen-rich water (saturated hydrogen in normal saline) can reduce malonaldehyde (MDA) activity and increase superoxide dismutase (SOD) activity, thereby reducing MIRI and improving cardiac function. At present, the mechanism of action of hydrogen is still in the exploration stage. In this study, a rat model of isolated heart ischemia-reperfusion was established, and a high-throughput G-Series Rat Cytokine Array 67 (GSR-CAA-67) protein chip was used to screen the differentially expressed proteins (DEPs) in the hydrogen-rich water group from the control group. Gene ontology (GO) enrichment and Kyoto Encyclopedia of Genes and Genome (KEGG) pathway enrichment analyses were used to summarize the biological processes and metabolic pathways that the DEPs are involved in, which provides a theoretical basis for the application of hydrogen-rich water in clinical settings.

## 2. Materials and Methods

### 2.1. Materials

#### 2.1.1Experimental animals

Twenty male Wistar rats, weighing 290-320g were provided by Beijing Vital River Laboratory Technology Co., Ltd. (Certificate of Conformity: SCXK (Beijing) 2016-0011). The present study was approved by the Animal Ethical and Welfare Committee of Hebei University (Baoding, China).

#### 2.1.2 Experimental reagents

The experimental hydrogen-rich water was provided by Mr. Zhilin Li from the School of Chemistry, Hebei University (the preparation technology has been granted a national patent, patent number ZL102557227B), G-Series Rat Cytokine Array 67kit (GSR-CAA-67, RayBiotech) was used.

#### 2.1.3 Laboratory apparatus

InnoScan 300 MicroArray Scanner (Innopsys) was used.

### 2.2 Methods

#### 2.2.1Establishment of myocardial ischemia-reperfusion model and groups

Twenty male Wistar rats were fed a normal chow diet for one week. The rats were randomly divided into a control group and a hydrogen-rich water group, with 10 rats in each group. Sodium pentobarbital (50 mg/kg) and heparin (250 U/kg) were injected intraperitoneally according to the body weight of the rats. After anesthesia was effectively administered, the abdominal wall was quickly opened from the lower rib of the xiphoid process, and the diaphragm was revealed. Simultaneously, the anterior line of the iliac crest was cut open and lifted up to the cephalad, without affecting the aorta of the heart. The heart was removed, the aorta was cannulated and fixed in a Langendorff device, and a 37°C perfusate with balanced oxygen (95% O_2_+5% CO_2_) was pre-perfused at a perfusion pressure of 7.85 kPa. The control group was perfused with K-R solution, and the hydrogen-rich water group was perfused with K-R solution + hydrogen-rich water (0.6 mmol/L, pH 7.3). After reverse perfusion for 10 min, the treatment was administered at room temperature for 20 min, and reperfusion was performed for 20 min. After completion, the rat left ventricular was collected.

#### 2.2.2Myocardial samples detected by protein chip

Rat myocardial tissue was first lysed and then quantified with a BCA assay. Finally, the sample was diluted to 500μg/ml. The slide chip was removed from the box equilibrated at room temperature for 20-30 min, the package was opened, the seal was removed, and the chip was placed in a vacuum desiccator or dried at room temperature for 1-2 h. One hundred microliters of sample dilution was added to each well and incubated for 1 h on a shaker at room temperature to block the semi-quantitative antibody chip. The buffer (500μg/ml loading) in each well was removed, and 100μl of the sample was added to the wells and incubated at 4℃ overnight. The slides were cleaned by Thermo Scientific Well wash Versa chip washer in two steps. First, 20× Wash I was diluted with deionized water, and the slides were washed 10 times with 250μl of 1× Wash I per well, oscillating for 10s with a high oscillation intensity. Then, dilute 20× wash solution II was diluted with deionized water, and the slides were washed six times with 250μl of 1× wash solution II per well, oscillating for 10s with a high oscillation intensity. The antibody mixture was centrifuged and added to 1.4 ml of the sample dilution, mixed well and centrifuged again quickly. Eighty microliters of the detection antibody was added to each well and incubated for 2 hours on a 37℃ shaker. Afterwards, the wells were washed. The Cy3-streptavidin tubule was centrifuged, and then 1.4 ml of the sample dilution was added, mixed well and centrifuge again quickly. Eighty microliters of Cy3-streptavidin was added to each well, and the slides were wrapped in aluminum foil to protect from light and incubated for 1 h on a 37℃ shaker. Afterwards, the wells were washed. The signal was scanned by the InnoScan 300 to detect fluorescence using Cy3 channel (excitation frequency = 532 nm). Data analysis was performed using GSR-CAA-67 data analysis software.

### 2.3 Statistical analysis

#### 2.3.1 Raw data normalization

The raw data obtained by the chip scan was subjected to chip background removal and inter-chip normalization processing by Raybiotech software.

#### 2.3.2 Screening for differentially expressed proteins

After the raw data were normalized by the software, the resulting data were selected for analysis. DEPs with P<0.05 were first retained and then further screened by Foldchange (expression difference multiple). The selection conditions were as follows: ①Foldchange≤0.83 or Foldchange ≥1.2; and ② Fluorescent signal > 150.

#### 2.3.3 Cluster analysis

For cluster analysis, the heatmap.2 function and *gplots* package from *R/bioconductor* were used. The distance between two samples was calculated as the Euclidean distance; the distance between the two clusters was calculated with the furthest neighbor method (complete), and the distance between classes was defined as the maximum distance.

#### 2.3.4 GO enrichment analysis of DEPs

Fisher's exact test and the *clusterProfiler* package from *R/bioconductor* were used. For selection, the number of genes that differed on a certain GO term/GO was ≥ 2, and P < 0.05.

#### 2.3.5 KEGG pathway enrichment analysis of DEPs

Fisher's exact test and the *clusterProfiler* package from *R/bioconductor* were used. For selection, the number of genes that differed on a certain term/pathway was ≥ 5, and P < 0.05.

## 3. Results

### 3.1 Establishment of DEPs profiles

Through the screening of DEPs in the control group and the hydrogen-rich water group, we obtained 25 DEPs (Table 1), and all DEPs in the hydrogen-rich water group (group C) were down-regulated compared with those in the control group (group H). By querying the information on these 25 proteins, most of them were found to be related to the inflammatory response 6.

### 3.2 Clustering Analysis of DEPs

**Figure 1:**
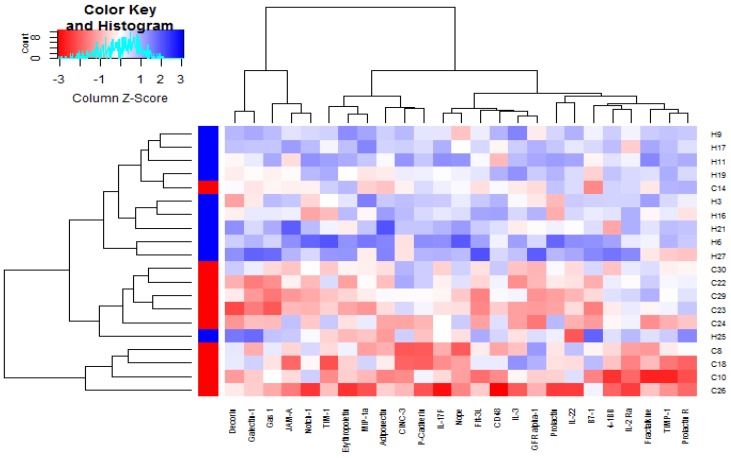
Clustering analysis of the differentially expressed proteins between group C and group H

By plotting the cluster heatmap, the global expression of these 20 samples and 25 DEPs can be presented from a macroscopic perspective. Figure 1 visually shows that the expression of DEPs in the control group (group H) and the hydrogen-rich water group (group C) are significantly different, and the DEPs in the hydrogen-rich water group are down-regulated relative to that in the control group.

### 3.3 GO Enrichment Analysis of DEPs

A total of 359 biological processes were screened in this experiment, and these processed involved signaling pathway regulation, immune response, and cardiovascular endothelial cell formation. Table 2 shows some of the results from this experiment (the biological processes in Table 2 are ranked in descending order of size according to the number of enriched genes (Count), taking the first 10 results). From Table 2, we found that six of these ten biological processes are related to the JAK-STAT pathway, namely, the STAT cascade, JAK-STAT cascade, regulation of the STAT cascade, regulation of JAK-STAT cascade, positive regulation of STAT cascade, and positive regulation of the JAK-STAT cascade.

### 3.4 KEGG Pathway Enrichment Analysis of DEPs

A total of 5 pathways were screened in this experiment. Table 3 shows the results from this experiment (the obtained pathways in Table 3 are sorted in descending order of magnitude according to the Count value).

## 4. Discussion

MIRI has a relatively complex mechanism, and its pathological process involves physiological mechanisms such as the inflammatory response, oxidative stress, and intracellular calcium overload 8-11. As a kind of element widely distributed in nature, hydrogen has a wide range of physiological effects such as anti-oxidation effects and the selective scavenging of free radicals 12. Presently, many scholars are studying the mechanism of hydrogen alleviation in ischemia-reperfusion injury tissues. Recent research found that hydrogen-rich water can reduce the expression of TNF-α during ischemia-reperfusion 13 and reduce myocardial infarct size 14. Gao Y et al 15 found that hydrogen-rich water can reduce endoplasmic reticulum stress, apoptosis and MIRI in rats. Presently, the protection mechanism of MIRI for hydrogen-rich water is still unclear.

High-throughput protein chips play an important role in scientific research due to its richer target factor detection and smaller sample volume requirement. It is widely used in the research of disease mechanisms 16-18. Recently, Bharath C 19 used the Raybiotech human AAH-APO-1 to study the molecular mechanism of cerebral ischemic apoptosis, and comprehensively revealed the changes of pro-apoptotic factors and anti-apoptotic expressed protein profiles in cerebral ischemia-reperfusion. This finding provides an important reference for further research on ischemic diseases. In this experiment, we used the GSR-CAA-67 protein chip from Raybiotech, USA, that can detect 67 proteins in rats. This protein chip was used to detect DEPs between the hydrogen-rich water group and the control group by constructing a rat isolated heart langendorff model. A total of 25 DEPs were screened out (P<0.05). Compared with their levels in the control group, the 25 DEPs in the hydrogen-rich water group were all down-regulated. This provides a basis for exploring the mechanism of action of hydrogen-rich water and finding a target for hydrogen-rich water.

Studies have shown that reducing inflammation can attenuate MIRI 20. Most of the 25 down-regulated proteins screened in this experiment were related to the inflammatory response, and it can be preliminarily concluded that hydrogen-rich water may protect the myocardium from damage by reducing the inflammatory response. The results of KEGG pathway enrichment analysis demonstrate that Th17 differentiation is related to the protective effect of hydrogen-rich water. Th17 cells are a subset of T cells associated with many inflammatory and autoimmune diseases that secrete inflammatory factors such as IL-17 and IL-22 21-22.There is data showing that Th17 cells are closely related to the occurrence of renal ischemia-reperfusion injury, and its expression is significantly increased in ischemia-reperfusion tissues 23. Table 1 and 3 show that the proteins involved in the differentiation of Th17 cells include IL-2Ra, IL-17F and IL-22, and the expression levels of these three proteins are decreased compared with those of the control group. Therefore, we speculate that the reason for the reduction of MIRI by hydrogen-rich water may be related to the inhibition of Th17 cell differentiation; however, the results need further verification.

By comparing the results of GO enrichment analysis and KEGG pathway enrichment analysis, we found that the JAK-STAT signaling pathway was enriched by both methods, indicating that the JAK-STAT pathway may be involved in the mechanism of action of hydrogen-rich water. The JAK-STAT signaling pathway has been studied in recent years, and it is widely involved in biological processes such as cell proliferation 24, differentiation 25, and the inflammatory response 26. The basic process of this pathway is as follows: cytokines bind to their corresponding receptors causing receptor dimerization → activation of JAKs → phosphorylation of STATs → STATs form dimers and enter the nucleus → STATs form dimers binding to the target gene promoter and then regulate gene expression 27. Some experimental studies have shown that the JAK-STAT pathway is closely related to myocardial ischemia-reperfusion, but its specific role is still unknown. Oh YB et al 28 constructed a rat isolate heart perfusion model and used a JAK3-specific blocker, JANEX-1, to block the JAK-STAT pathway and explore whether it would aggravate ischemia-reperfusion injury. They found that myocardial damage was significantly attenuated after blocking the JAK-STAT pathway, suggesting that activation of JAK-STAT is unfavorable for MIRI. However, a recent study had different results. Mudaliar H et al 29 found that remote ischemic preconditioning can attenuate early growth response-1 (EGR-1) expression, which is a key upstream activator in a variety of cardiovascular diseases, through the activation of the JAK-STAT pathway, thereby reducing MIRI. We have conducted in-depth research on the JAK-STAT 8 and PI3K-AKT pathway, and found that hydrogen-rich water can up-regulate the JAK-STAT and PI3K-AKT signaling pathway and alleviate MIRI in rats. The results showed that the P-JAK2/JAK2, P-STAT3/STAT3 and p-AKT/AKT increased when compared to the control, while the P-STAT1/STAT1 decreased in the hydrogen-rich water group. In addition, the apoptosis rate of the hydrogen-rich water group decreased significantly.

The GSR-CAA-67 protein chip used in this experiment can detect the expression level of only proteins, but there is no test for the modification of proteins. This requires in-depth research to be conducted in order to determine the target of hydrogen-rich water for MIRI.

## Figures and Tables

**Table 1:** The differentially expressed proteins between the case group and control group

Protein name	Gene ID	Foldchange (the group C/the group H)	*P*
Gas 1	683470	0.67366	0.000045
Flt-3L	103691134	0.66336	0.000088
IL-17F	301291	0.65264	0.000154
Galectin-1	56646	0.72155	0.000775
JAM-A	116479	0.70737	0.000943
TIM-1	286934	0.64563	0.028159
Adiponectin	246253	0.65508	0.002857
IL-3	24495	0.81244	0.003768
Erythropoietin	24335	0.75290	0.003970
4-1BB	500590	0.67767	0.005473
MIP-1a	25542	0.82481	0.006305
B7-1	25408	0.81611	0.006446
GFR alpha-1	25454	0.80775	0.009870
IL-2 Ra	25704	0.68152	0.011098
Notch-1	25496	0.77854	0.013427
Fractalkine	89808	0.68499	0.015341
IL-22	500836	0.59740	0.015728
P-Cadherin	116777	0.70260	0.017220
Decorin	29139	0.72011	0.020027
Nope	363081	0.76400	0.020463
CINC-3	114105	0.75123	0.024528
CD48	245962	0.82264	0.027651
TIMP-1	116510	0.64563	0.002634
Prolactin	24683	0.75411	0.036032
Prolactin R	24684	0.75411	0.045943

**Table 2:** The GO terms that are enriched with the differentially expressed proteins

GO terms	Gene Ratio^*^	Count^#^	*p*-adjust
cytokine-mediated signaling pathway	9/24	9	5.1772×10^-7^
response to molecule of bacterial origin	8/24	8	0.0000103
response to lipopolysaccharide	8/24	8	0.0000099
STAT cascade	7/24	7	5.7571×10^-7^
JAK-STAT cascade	7/24	7	5.75708×10^-7^
regulation of STAT cascade	6/24	6	0.0000099
regulation of JAK-STAT cascade	6/24	6	0.0000099
positive regulation of leukocyte proliferation	5/24	5	0.0002004
positive regulation of STAT cascade	5/24	5	0.0000099
positive regulation of JAK-STAT cascade	5/24	5	0.0000099

^*^ GeneRatio is the number of differential genes corresponding to GO term/the number of differential genes that can correspond to the same type in the GO database. ^#^ Count is the number of enriched genes.

**Table 3:** The KEGG pathway terms those are enriched with the differentially expressed proteins

KEGG pathway	Gene ID	Count^*^	*P*.adjust
Cytokine-cytokine receptor interaction	114105/89808/24684/500590/24335/103691134/25704/24495/301291/500836/25542/24683	12	2.00915×10^-12^
JAK-STAT signaling pathway	24684/24335/25704/24495/500836/24683	6	0.000037
Hematopoietic cell lineage	24335/103691134/25704/24495	4	0.001694
PI3K-Akt signaling pathway	24684/24335/25704/24495/24683	5	0.017927
Th17 cell differentiation	25704/301291/500836	3	0.027910

^*^Count is the number of enriched genes.

## Abbreviations

DEPs: differentially expressed proteins
MIRI: myocardial ischemia reperfusion injury
MDA: malonaldehyde
SOD: superoxide dismutase
GSR-CAA-67: G-Series Rat Cytokine Array 67
GO: Gene ontology
KEGG: Kyoto Encyclopedia of Genes and Genome
